# Aggressive Perioperative Management for Immune Thrombocytopenic Purpura in a Patient Undergoing Open Heart Surgery: A Correct Strategy in an Emergent Patient?

**DOI:** 10.7759/cureus.18310

**Published:** 2021-09-27

**Authors:** Hamdan Mallick, Sobia T Siddiqui, Yasir Khan, Ismael Ahmad, Syed Shahabuddin

**Affiliations:** 1 Medicine, The Aga Khan University Hospital, Karachi, PAK; 2 Cardiothoracic Surgery, The Aga Khan University Hospital, Karachi, PAK; 3 Internal Medicine, CMH Lahore Medical College and Institute of Dentistry, Lahore, PAK

**Keywords:** itp, cabg, cardiac surgery, itp managment, perioperative outcomes, combination therapy in itp

## Abstract

Immune thrombocytopenic purpura (ITP) is an autoimmune pathology that causes thrombocytopenia. This can become extremely troublesome when dealing with the rare clinical scenario of having to operate a highly invasive procedure on patients with thrombocytopenia. We report a case of a 66-year-old male with multiple comorbidities, including ITP, who underwent coronary artery bypass grafting (CABG) with an aortic valve replacement (AVR). He deteriorated rapidly, prompting urgent procedures. Little to no literature exists on the treatment plan for a critical patient with ITP who is about to undergo an open heart surgery. Our goal was to aggressively treat the patient with prednisolone, azathioprine, and platelets in the short preoperative time in order to maximize the prognosis. Our patient remained stable postoperatively, developed no complications, and was discharged successfully.

## Introduction

Immune thrombocytopenic purpura (ITP) is a condition that causes autoimmune destruction of platelets, increasing the predilection for bleeding and post-cardiac surgical complications [1,2]. We report a case of a 66-year-old male with multiple comorbidities, including ITP, who underwent coronary artery bypass grafting (CABG) with an aortic valve replacement (AVR) and was treated aggressively perioperatively for the emergent procedure. The recommended treatment for this rare clinical scenario includes packed platelets, intravenous immunoglobulin (IVIG), prednisolone, and splenectomy [1-5]. Perioperative management is key when facing an uncommon clinical scenario such as this. There is scarce literature on the relevant treatment regimens for the perioperative management of patients with ITP undergoing CABG. These regimens include aggressive perioperative strategies, such as multiple platelet infusions, steroids, and pharmacological therapy, in order to stabilize the patient and reduce the probability for peri- and postoperative complications.

## Case presentation

**Figure 1:**
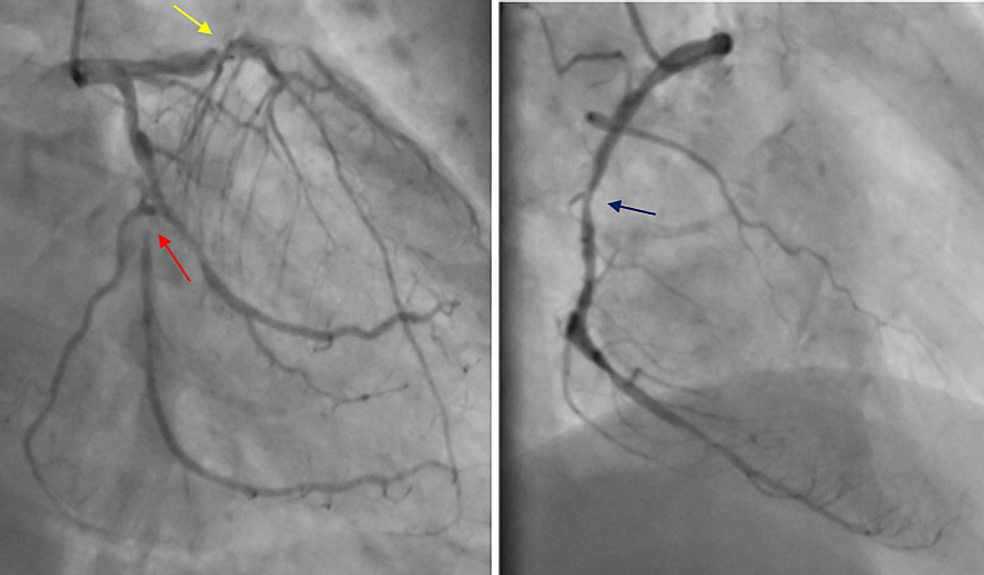
Coronary angiogram: triple-vessel occlusion Red arrow: 100% proximal obtuse marginal artery occlusion Yellow arrow: 80%–90% proximal left anterior descending artery occlusion Blue arrow: 80% right coronary artery occlusion

A 66-year-old male with a history of hypertension, diabetes mellitus, and hepatitis B (carrier) was admitted to the intensive care unit (ICU) with an acute ST-segment elevation myocardial infarction (STEMI). Immediate echocardiography revealed severe aortic valve stenosis, including a valve area of 1.2 cm^2^, moderate aortic and mitral regurgitation, and a left ventricular ejection fraction of 45%. Following this, a left heart cardiac catheterization was done, displaying a triple-vessel coronary artery disease with 80% occlusion of the mid-right coronary artery, 99% occlusion of the obtuse marginal artery, and diffuse disease of the left anterior descending artery (Figure 1) that warranted four coronary artery bypass grafting (CABG) with a single implanted aortic valve replacement (AVR). Laboratory investigations were standard, except for the severe thrombocytopenia with a 24 × 10^9^/L platelet count (normal count: 150-400 × 10^9^/L). Peripheral blood film displayed normocytic and normochromic red blood cells with low platelet levels. The finding of this severely low platelet count prompted a trephine biopsy, which showed a cellular marrow with trilineage hematopoiesis and normoblastic erythropoiesis. A presumptive diagnosis of immune thrombocytopenic purpura (ITP) was made on the basis of these findings. The patient's condition deteriorated quickly, and the procedure was scheduled with little time to significantly stabilize the platelet count. Preoperative patient optimization was done in collaboration with a multidisciplinary team, which included a hematologist. The goal remained to aggressively try and raise the platelet count while simultaneously treating the underlying ITP to minimize the risk of morbidity and intraoperative complications (Table 1). On this basis, the patient was given 1000 mg methylprednisolone daily for two days, including the surgery day, along with 50 mg oral azathioprine twice a day (BID). On the day of the surgery, blood sugar level was controlled with an insulin sliding scale, and the reported platelet count was 29 × 10^9^/L on complete blood count (CBC). The patient was then transfused with 10 units of platelets, four units of packed red blood cells, and four units of fresh frozen plasma two hours prior to the surgery. Then, 1000 mg each of methylprednisolone, ceftriaxone, and vancomycin was administered prior to skin incision, followed by intraoperative infusion of one unit of whole blood and six units of platelets. Postoperatively, 1000 mg of tranexamic acid was administered. Chest tube drainage was done, followed by timely extubation in the cardiac intensive care unit (CICU). The postoperative findings on the first day were unremarkable on clinical examination, but peripheral blood examination showed persistent thrombocytopenia with a platelet count of 32 × 10^9^/L, a white blood cell count of 9.5 × 10^9^/L (normal count: 4-10 × 10^9^/L), and hemoglobin of 14.2 g/dL (normal range: 13.7-16.3 g/dL). A consultation with the hematologist resulted in a decision to continue low-dose corticosteroids and a follow-up in two weeks. The total chest drain output was 350 mL in the CICU, and chest tubes were removed on the third postoperative day (POD). The postoperative course was uneventful, and the patient was discharged on the eighth POD. Peripheral blood examination on discharge still showed thrombocytopenia with a platelet count of 44 × 10^9^/L, a white blood cell count of 8.3 × 10^9^/L, and hemoglobin of 15.1 g/dL.

**Table 1:** Aggressive management at each operative stage

Operative Stage	Management and Intervention		Platelet Count
Preoperative (Day 1 & 2)	1000 mg methylprednisolone and 50 mg oral azathioprine BID		24 x 10⁹/L
Intraoperative (Day 2)	2 hours prior to incision	10 units of platelets, 4 units of packed red blood cells, 4 units of fresh frozen plasma, and 1000 mg of methylprednisolone	29 x 10⁹/L
	During operation	1 unit of whole blood transfusion and 6 units of platelets	
Postoperative (Day 2 & 3)	1000 mg tranexamic acid		32 x 10⁹/L

## Discussion

ITP is an autoimmune pathology resulting from the destruction of platelets by autoantibodies [1-4,6-9]. Thus far, however, complex mechanisms involving impaired platelet production, T-cell-mediated effects, and relative marrow failure have also been proposed [2,3]. Thrombocytopenia resulting from ITP is not protective against acute coronary syndrome, as reported by previous cases [2,6]. This may be due to the coronary thrombosis resulting from abnormal platelet size and increased adhesiveness to the vascular surface or the activation of inflammation and coagulation cascade [2]. A separate theory hypothesized that damage to the endothelial cells caused by autoantibodies that are directed at antigens present on both coronary endothelial cells and platelets may result in myocardial infarction in patients with thrombocytopenia with ITP [7]. In a rare case where a patient with ITP does develop an acute coronary syndrome, prior risk factors, such as diabetes, hypertension, and a hypercoagulable state, should be looked at [7]. Our patient had preexisting hypertension, diabetes mellitus, and hepatitis B. These comorbidities may have added and contributed to the development of acute coronary syndrome. In the largest sample from a report in literature by Jubelirer et al., mild or moderate cases of thrombocytopenia were significantly supported by intravenous immunoglobulin (IVIG) and platelet transfusions. The role of preoperative short-term prednisone therapy was not helpful in this sample [4]. The role of preoperative prednisone is less conflicted in other studies where its use as a treatment option was reported to be successful as initial treatment [1,3,4]. However, it is important to note that the role of prednisone is most advantageous when it is given over a period of days to weeks in order to allow the platelet count to stabilize [3,4]. In our case, the patient required emergent CABG due to triple-vessel disease. This made the preoperative management quite difficult as a multiple drug regime was needed to prepare the patient for an emergent operation that would not allow enough time for stabilizing the preoperative platelet count. Platelet infusions can be preferred here when a rapid increase in the platelet count is desired [3]. More studies, however, are needed to determine the real effectiveness of prophylactic platelet transfusions [6]. IVIG has also been a viable option in patients refractory to steroid treatment and is even suggested as a possible preoperative treatment of choice [2,9]. The real usefulness of IVIG is in its rapid action [2,3,8]. However, in a lower-middle-income setting such as ours, the availability and cost play against the use of this specific therapy [1]. Splenectomy has been suggested as a second-line treatment option in patients resistant to IVIG therapy. However, this is a high risk procedure and requires several days for an appropriate response [1,9]. Cardiopulmonary bypass (CPB) induced platelet dysfunction and the use of anticoagulants postoperatively may put the patient at a higher risk of cardiac tamponade during the immediate and late phase of recovery [8]. This is made worse by the thrombocytopenia as a result of ITP. Hence, the management options of such a patient become extremely subjective and difficult. Excellent response was seen in our patient with preoperative treatment of methylprednisolone, azathioprine, and aggressive transfusion of platelets for raising the platelet count. As recommended by the American Society of Hematology, careful monitoring of the patient's hemodynamic condition was conducted as he was treated with prednisolone while predisposed to hypertension [5]. In patients such as this, life changes may be beneficial. Extra precaution will now need to be taken, given the age of the patient and the probable further treatment with corticosteroids. Importantly, even with platelet counts of up to 50 × 10^9^/L, the American Society of Hematology reports benefit in corticosteroid therapy as compared to routine patient observation [5]. Hypertension, hepatitis, and a history of acute coronary syndrome make monitoring of ITP and geriatric care very imperative, especially given the patient's age and history of extensive comorbidities. An established relationship and consults with a primary care physician, following a complete breakdown of the patient's condition, may allow for motivation for increased routine checkups, labs, and overall patient satisfaction.

## Conclusions

Tranexamic acid, azathioprine, steroids, and platelet transfusions can be used for perioperative management of patients with thrombocytopenia caused by ITP. This aggressive management protocol may reduce the need for transfusion and reexploration requirements without burdening the risk for serious adverse reactions postoperatively. The use of intravenous immunoglobulin therapy may be restricted in resource- and time-limited cases. Focusing on an aggressive preoperative treatment regimen will decrease the risk associated with a low and unstable platelet count in an emergent open heart surgery and may also lower postoperative morbidity and complications. In geriatric patients such as our case, extra precautions will have to be taken in order to decrease the risk for future adverse patient outcomes.
